# Immunologic Targeting of FOXP3 in Inflammatory Breast Cancer Cells

**DOI:** 10.1371/journal.pone.0053150

**Published:** 2013-01-14

**Authors:** Smita Nair, Amy J. Aldrich, Eoin McDonnell, Qing Cheng, Anshu Aggarwal, Pujan Patel, Monique M. Williams, David Boczkowski, H. Kim Lyerly, Michael A. Morse, Gayathri R. Devi

**Affiliations:** 1 Department of Surgery, Duke University Medical Center, Durham, North Carolina, United States of America; 2 Duke Cancer Institute, Duke University Medical Center, Durham, North Carolina, United States of America; 3 Department of Medicine, Duke University Medical Center, Durham, North Carolina, United States of America; Université Paris Descartes, France

## Abstract

The forkhead transcription factor FOXP3 is necessary for induction of regulatory T lymphocytes (Tregs) and their immunosuppressive function. We have previously demonstrated that targeting Tregs by vaccination of mice with murine *FOXP3* mRNA-transfected dendritic cells (DCs) elicits FOXP3-specific T cell responses and enhances tumor immunity. It is clear that FOXP3 expression is not restricted to T-cell lineage and herein, using RT-PCR, flow cytometry, and western immunoblot we demonstrate for the first time that FOXP3 is expressed in inflammatory breast cancer (IBC) cells, SUM149 (triple negative, ErbB1-activated) and SUM190 (ErbB2-overexpressing). Importantly, FOXP3-specific T cells generated *in vitro* using human FOXP3 RNA-transfected DCs as stimulators efficiently lyse SUM149 cells. Interestingly, an isogenic model (rSUM149) derived from SUM149 with an enhanced anti-apoptotic phenotype was resistant to FOXP3-specific T cell mediated lysis. The MHC class I cellular processing mechanism was intact in both cell lines at the protein and transcription levels suggesting that the resistance to cytolysis by rSUM149 cells was not related to MHC class I expression or to the MHC class I antigen processing machinery in these cells. Our data suggest that FOXP3 may be an effective tumor target in IBC cells however increased anti-apoptotic signaling can lead to immune evasion.

## Introduction

Forkhead box protein 3 (FOXP3), a member of the forkhead winged helix family of transcriptional regulators is a nuclear protein expressed in regulatory T cells (Tregs) and plays a critical role in regulating the development and immunosuppressive function of Tregs [1], [2]. Despite an essential role in preventing autoimmunity, prevalence of Tregs is increased in the blood and the tumor microenvironment of patients with a variety of different tumors, including breast cancer, relative to healthy subjects suggesting a role of Tregs in suppressing anti-tumor immune responses [3]–[14]. Indeed, since FOXP3 Tregs are immunosuppressive cells, many studies have reported that their abundant presence in tumor infiltrates leads to reduced survival in cancer patients. Also, clinical response of breast cancer to therapy is associated with reductions in Tregs [12]. Ladoire et al [15] reported that a complete histological response to neoadjuvant breast cancer chemotherapy was associated with absence of intratumoral FOXP3 cells. Recently, we observed that use of a FOXP3 targeting antisense morpholino oligomer to deplete Tregs resulted in enhanced generation of antigen-specific T cells in response to peptide stimulation in peripheral blood mononuclear cells [16].

Despite a clear role for FOXP3 in Tregs, FOXP3 protein expression is not restricted to the lymphocyte lineage but is also present in cancer cells of non-hematopoietic origin [13], [17]–[19]. In pancreatic cancer and melanoma, FOXP3 expression was restricted to tumor cells and the normal pancreatic ducts or melanocytes were devoid of FOXP3 expression. Niu et al suggest that FOXP3 expression in melanoma cells renders the cells suppressive with Treg-like activity such that FOXP3 expressing melanoma cells directly inhibit the proliferation of T cells and may represent a possible mechanism of tumor resistance to immune destruction in the melanoma tumor microenvironment [20]. The expression pattern and role of FOXP3 in breast cancer has been more difficult to elucidate. Zuo et al [18], [21] demonstrated that FOXP3 is an X-linked breast cancer suppressor gene and an important regulator of the epidermal growth factor receptor (HER2/ErbB2) oncogene. They also reported that FOXP3 is a novel transcriptional repressor for the oncogene SKP2 in breast cancer cells that do not overexpress HER2/ErbB2 [21]. *FOXP3* also induces expression of several tumor suppressors including p18 (CDKN2C), p21 (CDKN1A), LATS2, and ARHGAPS [22]. It binds to and negatively regulates the activity of NF-κB and IL-2 [13], [23], [24]. However, FOXP3 expression does not show a clear differential pattern in breast cancer cells and several reports have also shown that FOXP3 expression correlates with unfavorable prognosis in breast cancer [25]–[28]. Further, FOXP3 expression in inflammatory breast cancer (IBC), an aggressive subtype of breast cancer with the worst survival outcome amongst all breast cancers [29], [30] has not been established. Therefore, in the current study we evaluated FOXP3 expression in IBC cells. In addition, we studied its role as a possible antigenic target in SUM149, a cellular model for basal-type IBC and its isogenic derived cell line-rSUM149 cells [31] with acquired therapeutic resistance to lapatinib, an epidermal growth receptor (EGFR/HER2) dual kinase inhibitor approved for use in IBC patients. We have previously demonstrated that FOXP3 is an effective immunotherapeutic target, and vaccination of mice with murine FOXP3 mRNA-transfected dendritic cells (DCs) elicits FOXP3-specific T cell responses and enhances tumor immunity [32]. We therefore used the human FOXP3 RNA transfected DCs to stimulate autologous FOXP3-specific T cells and used them as effector cells to determine FOXP3-targeting potential in IBC cells.

## Materials and Methods

### Cell Lines

SUM149 and SUM190 cells were obtained from Asterand, Inc. (Detroit, MI). rSUM149 is an isogenic-derivative of SUM149 selected for resistance to lapatinib-analog, GW583340 and maintained in 7.5 µmol/L GW583340-containing media as described previously [33]. SKBR3 (human adenocarcinoma of the breast, pleural effusion), MCF-7 (human adenocarcinoma of the breast, pleural effusion), and BT474 (human ductal carcinoma) cells were obtained from American Type Culture Collection (ATCC) and cultured as previously described [34]. Jurkat E-6 (pseudodiploid acute T-cell leukemia) cells were obtained from ATCC and cultured in RPMI-1640 media (Gibco) with 10% FBS and pencillin/streptomycin. 293T (human embryonic renal epithelial) cells were obtained from ATCC and cultured in Dulbecco’s Modified Eagle’s medium (DMEM), with 10% FBS and 1% penicillin/streptomycin. The cells were cultured in an incubator at 37 degrees with 5% CO2 and passaged every 2–3 days. HME-1 cells (immortalized human mammary epithelial, ATCC) were cultured in MEBM media (Lonza) with bullet kit additives (Lonza), 10% FBS and 1% pencillin/streptomycin. Peripheral blood mononuclear cells (PBMCs) and subsequent extraction of human monocytes, and Tregs from buffy coats of healthy, normal donors were prepared as previously described [35].

### Ethics Statement

Primary human cells used in these experiments were isolated from leukapheresis products that were obtained from human subjects following written informed consent using protocol (Pro00012388) approved by the Duke University Institutional Review Board.

### Gene Expression Data Analysis

We compiled a collection of 4010 breast tumor gene expression data derived from 23 datasets that have been posted on the NCBI Gene Expression Omnibus (GEO) database, as previously described [36]. In addition to the raw expression data, we also obtained recurrence-free survival data from a subset of the samples (n = 1372). Average expression signal of probe sets 221333_at and 221334_s_at was used to represent *FOXP3* expression. We assigned each of 4010 sample into Low (first and second quartiles, lower 75%) and High (third quartile, highest 25%) subgroups according to *FOXP3* expression levels, and compared prognosis differences between these two subgroups in different breast cancer subtypes, using Kaplan-Meier Estimates of recurrence-free survival analysis.

### Flow Cytometry

Cells were cultured in six-well plates (Corning Incorporated, Corning, NY) in regular growth media until they reached 70–80% confluence. Cells were fixed, permeabilized, and washed. FOXP3 was detected using the PE-conjugated anti-FOXP3 staining set (BD Biosciences, clone PCH101 for human samples) and matched isotype control rat antibody (BD Biosciences). Flow cytometric analysis was performed on the Becton Dickinson LSR caliber and the data was analyzed using the FACSDiva v6.1.1 software. Each histogram represents the cell line FOXP3 peak compared to that of rat IgG peak.

### Protein Expression

Cells were harvested and immediately lysed in NP40 cell lysis buffer (BioSource) with fresh protease inhibitor cocktail (Sigma) and 1 mmol/L phenylmethylsulfonylfluoride. Protein concentration was determined by the Pierce BCA Protein Assay Kit. Equal amounts of cell lysates were then subjected to SDS-PAGE under reducing conditions. Before loading onto the gel, all lysates were boiled for 5 min and immediately cooled on ice. The protein was transferred onto Immobilon-P membrane (Millipore) previously soaked in methanol and transfer buffer by the TRANS-BLOT SD semidry transfer cell (Bio-Rad). After the transfer process was complete, the membranes were incubated with blocking buffer (5% dry nonfat milk in 1X TBS-0.1% Tween 20) for 1 hour at room temperature. Membranes were incubated in primary antibody solution containing FOXP3 (1∶250; PCH101; eBioscience; San Diego, CA), SKP2 (1∶1000; Cell Signaling; Danvers, MA), p27 (1∶1000; Cell Signaling; Danvers, MA), XIAP (1∶1000; BD Biosciences; San Jose, CA) overnight at 4 degrees or GAPDH (1∶4,000; Santa Cruz Biotechnology, Santa Cruz, CA) for one hour at room temperature. After washing with buffer containing PBS and Tween 20, membranes were incubated in a secondary antibody solution containing the appropriate secondary antibody conjugated to horseradish-peroxidase (1∶2000) for one hour. After washing, membranes were subjected to SuperSignal West Pico Chemiluminescent substrate reagents (Pierce). Bands were visualized using the Kodak BioMax XAR X-ray film.

### Immunofluorescence

Breast cancer cells were seeded onto sterile 10×10 mm coverslips in 6-well plates, and then allowed to grow and attach for 24 hours. Cells were fixed onto the coverslips by submersion in paraformaldehyde for 15 minutes. The cells were washed for 5 minutes three times in PBS containing 0.1% BSA and incubated in blocking buffer consisting of 10 mL PBS, 0.25% Triton X-100, 100 mg BSA, and 500 µL normal goat serum (Jackson Laboratories) overnight at 4°C. Primary and secondary antibodies were dissolved in blocking buffer. The cells were incubated in primary rat anti-human antibody solution (FOXP3, 1∶50; PCH101, eBioscience) for 1 hour at room temperature. The cells were washed 2 times for 5 minutes in PBS containing 0.1%BSA and incubated with a 1∶150 diluted fluorescently tagged secondary antibody, Alexa Fluor® 568 goat anti-rat IgG (Molecular Probes) for 1 hour in the dark at room temperature. The cells were washed twice for 5 minutes and the coverslips were carefully removed from the culture plates and dipped into water for 2–3 seconds and mounted with ProLong Gold anti-fade reagent with DAPI (Invitrogen) onto slides. To prevent the escape of the mounting medium, coverslips where sealed around the edges. The slides were kept in the dark at 4°C until pictures were taken using the Zeiss Axio Observer and MetaMorph 7.5 systems.

### Cloning of Human FOXP3

Total RNA was isolated from human CD4+/CD25+ Tregs. Reverse transcription was primed with oligo dT and PCR was conducted using the following primers:

*5′ TATATAAAGCTTGCCACC* ***A*** *TGGCCCTTGGCCCAT-3′.*

*5′-TATATAGGATCCTCAGGGGCCAGGTGTAGGGTTG-3′.*

The resulting 1256 bp fragment was cloned into the HindIII and BamHI sites of pSP73-Sph/A64, which has a T7 promoter 5′ to the insertion site and 64 adenine nucleotides 3′ to the insert that allow for the production of *in vitro* transcribed RNA with a polyA tail of 64 residues. Successful sub-cloning of FOXP3 was confirmed by DNA sequencing.

### *In vitro* Transcription of RNA

The plasmids were digested with the restriction enzyme SpeI to linearize the DNA followed by *in vitro* transcription using the T7 mMessage Machine (Ambion, Austin, TX). The *in vitro* transcribed (IVT) RNA was purified using a commercial kit (RNA Easy, Qiagen, Valencia, CA), quantified by spectrophotometry, and analyzed by MOPS formaldehyde gel electrophoresis to confirm the synthesis of full-length RNA.

### Generation of Human Dendritic Cells (DCs)

Peripheral blood mononuclear cells (PBMCs) were thawed, washed in PBS and resuspended at 2×10^8^ cells in 30 ml AIM-V media (Invitrogen) in T-150 tissue culture flasks. Cells were incubated for 1 hour at 37°C, 5% CO_2_ in a humidified incubator. The non-adherent cells were harvested by rocking the flask from side to side to dislodge them. The adherent cells were replenished with 30 ml AIM-V media supplemented with 800 U/ml human GM-CSF and 500 U/ml human IL-4 and then incubated at 37°C [37]. DCs were harvested on day 6, by collecting all non-adherent cells, followed by cold PBS wash. DCs that were still adherent were dissociated with cell dissociation buffer (Invitrogen), 37°C for 20 minutes. DCs were washed, counted and maintained on ice until use.

### Transfection of DCs with RNA Encoding FOXP3

DCs were washed two times and gently resuspended in Opti-MEM at 2.5–3×10^7^/ml. 100–200 µl were transferred to a 2-mm cuvette for electroporation using an Electro Square Porator ECM 830 from BTX [38]. 3–4 µg RNA per 10^6^ cells was added to the cuvette and mixed by pipetting up and down gently but thoroughly. The cells and RNA were pulsed at 300 Volts for 500 microseconds. The efficiency of RNA transfection was assessed routinely using RNA encoding green fluorescent protein (GFP) [39] followed by flow cytometric analysis (data not shown). Transfected DCs were immediately transferred to plates containing media at 10^6^ cells/ml and incubated in media containing the maturation cytokine cocktail at 37°C, 5% CO_2_ for 6–7 hours. Maturation media is AIM-V containing GM-CSF (800 U/ml), IL-4 (500 U/ml), TNF-α (5 ng/ml), IL-1β (5 ng/ml), IL-6 (150 ng/ml) and PGE_2_ (1 µg/ml). All cytokines are obtained from Peprotech Inc. PGE_2_ is purchased from Sigma.

### Stimulation of T cells with RNA-transfected DCs *in vitro*

The ability of human DCs transfected with antigen (or control)-encoding RNA to stimulate CTL cell responses *in vitro* was determined [38]. PBMCs were thawed, washed, resuspended in PBS and treated with DNase I (Sigma) at 200 U/ml for 20 minutes at 37°C. DNase I-treated PBMCs were used to isolate T cells using the EasySep T cell Enrichment Kit (StemCell Technologies, Vancouver, BC), for negatively isolating CD3^+^ T cells. The isolated CD3^+^ T cells were stimulated with RNA-transfected, matured DCs at a responder to stimulator ratio (R:S) of 10∶1 in the presence of 25 ng/ml IL-7 at 37°C. All stimulations were performed in RPMI 1640 with 10% FCS, 2 mM L-glutamine, 20 mM HEPES, 1 mM Na-pyruvate, 0.1 mM MEM non-essential amino acids, MEM amino acids (50×solution), 100 IU/ml penicillin, 100 µg/ml streptomycin and 5×10^−5^ M ß-mercaptoethanol (CTL stimulation medium). The responder T cell concentration was 2–2.5×10^6^ cells/ml. IL-2 was added on day 3 at 100 U/ml. On day 7, the T cells were restimulated with RNA-electroporated DCs at a R:S of 10∶1 in complete RPMI-10% FCS in the presence of 50–100 U/ml IL-2, with the T cells at 1–2×10^6^ cells/ml in CTL stimulation medium. After 5–6 days the T cells were harvested, counted and used as effector cells in a europium-based CTL assay.

### IFN-γ Secretion

FOXP3-specific T cells were generated as described above. T cells were allowed to rest overnight in RPMI medium with 10% FCS in the absence of cytokines. The following day, T cells were harvested and cocultured with target tumor cells (SUM149, rSUM149 and 293T cells) at a 1∶1 ratio. T cells and target cells were maintained at 10^6^ cells/ml and a 100 µl of each cell suspension was added to a 96-well V-bottom tissue culture plate in triplicates and incubated at 37°C for 16 hours. Supernatant was harvested and IFN-γ secretion was measured using a human IFN-γ ELISA kit from eBioscience.

### Target Cells for T cell Cytotoxicity Assays

Tumor cells or autologous DCs transfected with RNA were used as targets. DCs were transfected with RNA and incubated overnight in GMCSF+IL-4 AIM-V media prior to europium labeling for use as target cells.

### *In vitro* Cytotoxicity Assay

5–10×10^6^ target cells were labeled with europium for 20 minutes at 4°C using a standard published procedure [40], [32]. 10^4^ europium-labeled targets and serial dilutions of effector cells at varying E:T ratio as indicated in the figures were incubated in 200 µl of CTL medium (CTL stimulation medium with no penicillin-streptomycin) in 96-well V-bottom plates. The plates were centrifuged at 500×g for 3 minutes and incubated at 37°C for 4 hours. 50 µl of the supernatant was harvested and added to 150 µl of enhancement solution (Wallac, Perkin-Elmer) in 96-well flat-bottom plates and europium release was measured by time resolved fluorescence using the VICTOR3 Multilabel Counter (Perkin-Elmer) [40], [32].

Specific cytotoxic activity is determined using the formula: % specific release = [(experimental release - spontaneous release)/(total release - spontaneous release)]×100. Spontaneous release of the target cells was less than 25% of total release by detergent in all assays for the results of the assay to be valid. Spontaneous release of the target cells was determined by incubating the target cells in medium without T cells. All assays were performed in triplicates.

### Reverse Transcriptase PCR

Total RNA was isolated using the Trizol Reagent (Sigma, St. Louis, MO), total RNA Aurum isolation kit (Bio-rad), and/or RNA-easy mini kit (Qiagen) according to the manufacturer’s instructions. The supplier’s instructions in the iScript cDNA synthesis kit (Bio-Rad) were followed to reverse-transcribe using 2 µg RNA. Each PCR reaction mixture contained PCR Master Mix (12.5 µL; Promega, Madison, WI), cDNA (2 µl), forward primer (2.5 µL; 1 mM), reverse primer (2.5 µL; 1 mM), and RNase/DNase Free Water (5.5 µL). The PCR reaction mixtures were placed in the PCR machine heat block and the cDNA was initially denatured at 95°C for 15 minutes. PCR amplification was performed by denaturation for 60 seconds at 94°C, annealing for 60 seconds at 66°C, primer extension for 60 seconds at 72°C, and a final extension for 10 minutes at 72°C. The primer sequences are as follows [13]:

*FOXP3 Fwd 5′ CACAACATGCGACCCCCTTTCACC-3′,*

*FOXP3 Rev5′AGGTTGTGGCGGATGGCGTTCTTC-3′,*

*ß-actin Fwd: 5′CCTTCTACAATGAGCTGCGTGTG-3′,*

*ß-actin Rev: 5′GAGGCGTACAGGGATAGCACAG-3′.*

### qRT-PCR

RNA was isolated using either the total RNA Aurum isolation kit (Bio-Rad) or RNAeasy mini kit (Qiagen). 1 µg RNA was used in each 20 µl reverse transcription reaction using the Bio-Rad iScript cDNA synthesis kit. The cDNA was then diluted 1∶10. Expression levels of antigen presentation machinery (APM) were analyzed by qPCR. Each qPCR was performed using Bio-Rad SYBR green supermix with 0.2 µM of both forward and reverse primers and 2.25 µl diluted cDNA for a total reaction volume of 6.5 µl. Amplification was carried out using the Bio-Rad CFX384 qPCR system. Data was analyzed using the ΔΔC(t) method using GAPDH as the normalization control, and represented as relative expression (2^−ΔΔC(t)^). Samples were analyzed in triplicate. Sequences are as follows:

*FOXP3 spanning exons 1,2 Forward: 5′-ACAGCACATTCCCAGAGTTC-3′,*

*Reverse: 5′-CCAGTGGTAGATCTCATTGAGTG-3′;*

*FOXP3 spanning 3,4 Forward: 5′-AACCCCATGCCACCATCGCA-3′,*

*Reverse: 5′-TCCACCGTTGAGAGCTGGTGC-3′;*

*TAP-1 Forward: 5′-AGAAGGTGGGAAAATGGTACC-3′,*

*Reverse: 5′-GTTGGCAAAGCTTCGAACTG-3′;*

*TAP-2 Forward: 5′-GTGTGATTGACATCCTGGGAG-3′;*

*Reverse: 5′-TCCGCAAGTTGATTCGAGAC-3′.*

*LMP-2 Forward: 5′-GAGAGGACTTGTCTGCACATC-3′,*

*Reverse: 5′-GCATCCACATAACCATAGATAAAGG-3′;*

*LMP-7 Forward: 5′-CCTACATTAGTGCCTTACGGG-3′,*

*Reverse: 5′- TCCATTTCGCAGATAGTACAGC-3′.*

*GAPDH Forward: 5′-ATCCCATCACCATCTTCCAGGAG-3′,*

*Reverse: 5′-CAAATGAGCCCCAGCCTTCTC-3′.*

### IFN-γ Treatment

SUM149 and rSUM149 cells were plated in separate 6 well plates at a density of 7.5×10^4^ cells/well. After 24 hours media was replaced and the appropriate wells were treated with IFN-γ (20 ng/ml) for a period of 20 hours. Expression levels of antigen presentation machinery (APM) were analyzed by qPCR as described above.

### Statistical Analysis

The statistical analyses were performed using Graphpad InStat (Graphpad Software, Inc., La Jolla, CA) Student’s two-tailed t-test. Differences were considered significant at p<0.05.

## Results

### FOXP3 is Expressed in Inflammatory Breast Cancer Cells

**Figure 1:**
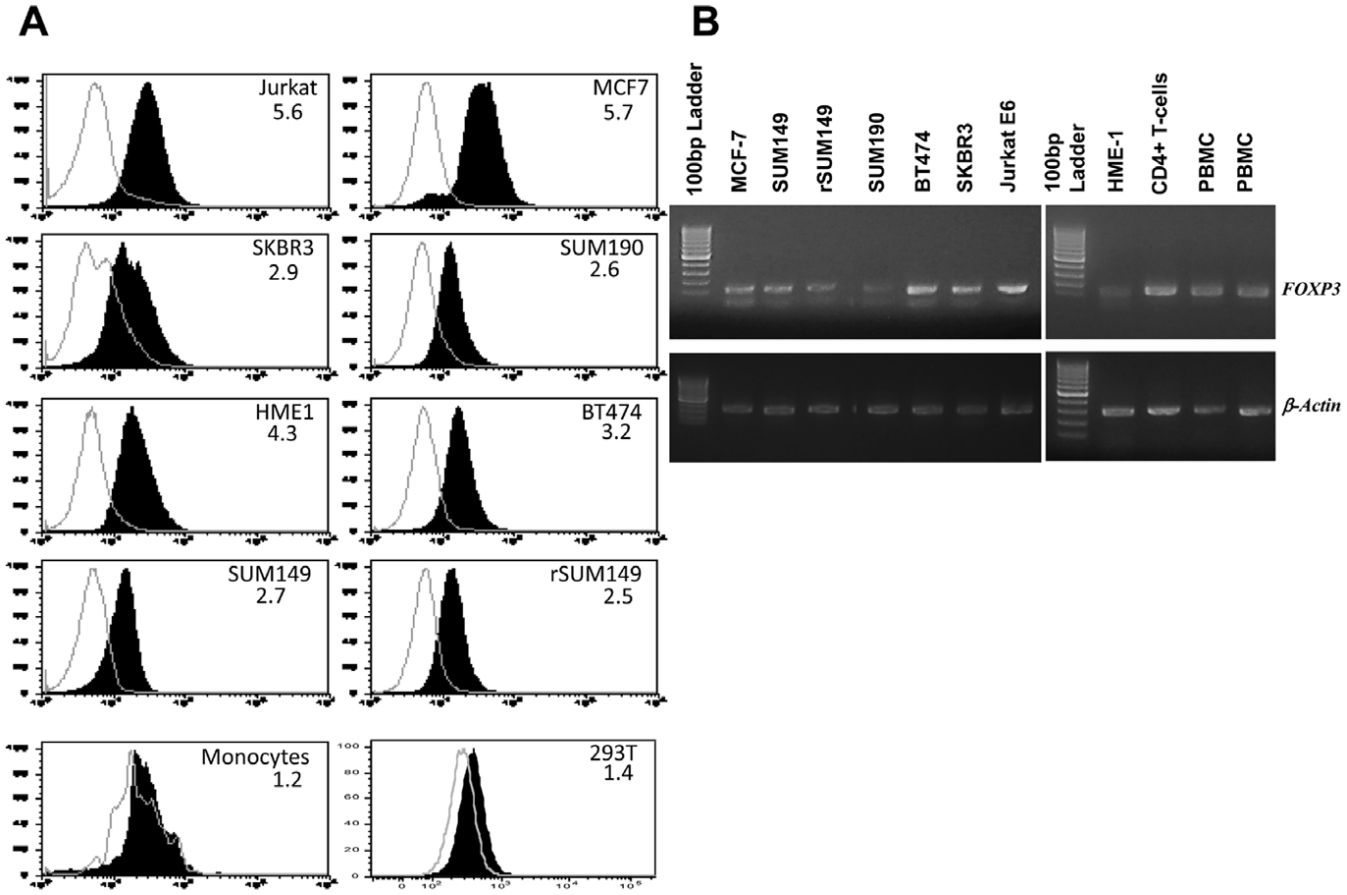
FOXP3 expression in IBC cells. A. Flow cytometry-based detection of FOXP3 expression in various cell lines. Monocytes (high Side Scatter (SSC), high Forward Scatter (FSC) cells within human PBMCs) were used as a negative control for FOXP3 expression. Each histogram represents the cell line FOXP3 peak compared to that of isotype peak (white underlaid plot). The mean fluorescence intensity (MFI) ratio (FOXP3/isotype) is shown in each histogram. **B.** Expression of FOXP3 mRNA by RT-PCR in HME1, breast cancer cells (MCF-7, BT474, SKBR3), IBC cell lines (SUM149, SUM190, rSUM149), Jurkat, human PBMCs, CD4+ T cells isolated from human PBMCs. Actin mRNA is used to verify the integrity of the cDNA preparations.

To evaluate FOXP3 expression in IBC cells, we examined SUM149, a triple negative, basal type and SUM190, an ErbB2 overexpressing cell line, both of which are well established models isolated from IBC patient tumors, rSUM149 isogenic-derivative of SUM149 and compared them to non-IBC breast cancer cells (MCF-7, BT474, SKBR3), human mammary epithelial cells (HME1), Jurkat-E6, freshly isolated normal PBMCs, human monocytes and CD4+ T cells [31], [33]. FOXP3 protein expression by flow cytometric analysis was clearly detected (Figure 1A) in the SUM149, rSUM149 and SUM190 cells and insignificant expression in 293T cells. MCF7 and Jurkat were used as positive controls, and monocytes as negative controls for FOXP3 expression as reported previously [41]. Each histogram in Figure 1A represents the cell line FOXP3 peak compared to that of isotype rat IgG peak. FOXP3 RNA expression detected by qualitative PCR reveals a 167 bp band specific to FOXP3, as verified by sequence analysis (Figure 1B).

### FOXP3-specific CTL Lyse Human IBC Cell Line SUM149

**Figure 2:**
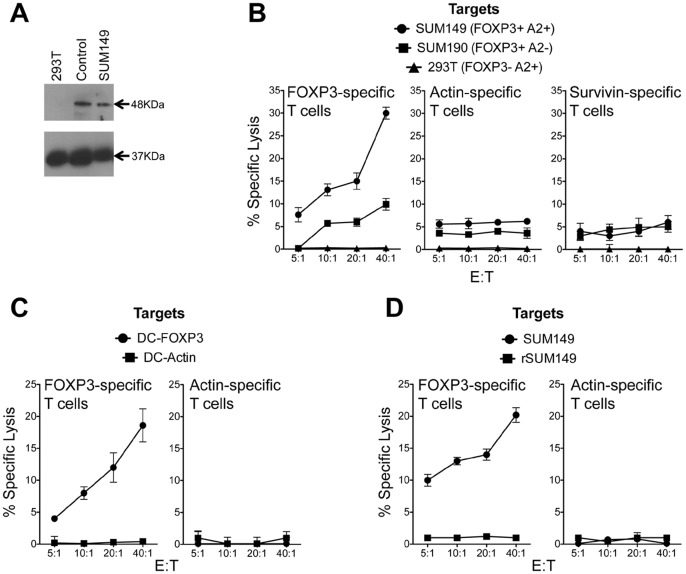
Lysis of IBC cell line, SUM149 by FOXP3-specific T-cells stimulated with FOXP3-encoding RNA-transfected DCs. A. Immunoblot analysis of 48 kDa FOXP3 and 37 kDa control GAPDH protein expression in 293T, SUM149 cell lysates and tonsil tissue lysates. **B.** Non-adherent PBMCs and DCs were generated from cells obtained from HLA-A2+ healthy donors. DCs were transfected with FOXP3 RNA, survivin RNA and actin RNA and used to stimulate autologous T cells as described in Methods. Post-stimulation the T cells were assayed for lytic activity using a europium-release assay. T cell lytic activity was measured against IBC cells SUM149 and SUM190 and 293T cells as controls. **C.** DCs expressing FOXP3 and actin were used as targets to demonstrate specificity using effector T cells described in 2A. **D.** FOXP3-specific T cell cytolytic activity on SUM149 and lapatinib-resistant, rSUM149 cells. Non-adherent PBMCs and DCs were generated from cells obtained from HLA-A2+ healthy donors. DCs were transfected with FOXP3 RNA and actin RNA and used to stimulate autologous T cells as described in Methods. Post-stimulation the T cells were assayed for lytic activity using a europium-release assay. T cell lytic activity was measured against IBC cells SUM149 and rSUM149 cells.

We have previously demonstrated that vaccination of mice with FOXP3 mRNA-transfected DCs elicits FOXP3-specific T cell responses and enhances tumor immunity [32]. Moreover this enhancement in anti-tumor immunity is associated with elimination of FOXP3-expressing intra-tumoral Tregs. Since FOXP3 is expressed by IBC cells, we wanted to evaluate if targeting FOXP3 for immunotherapy is a viable strategy. Human FOXP3 was cloned from human CD4+CD25+ Tregs and used to generate FOXP3 mRNA. PBMCs from an HLA-A2+ donor were used to generate DCs and T cells. Monocyte-derived DCs were transfected with FOXP3 mRNA and used to stimulate autologous T cells *in vitro*. As control antigens we used DCs transfected with human survivin mRNA [42] and human actin mRNA [40] to stimulate T cells. Effector T cells generated after two rounds of *in vitro* stimulation were used in a cytolytic assay to measure T cell effector function. As targets we used the FOXP3-expressing IBC cell lines SUM149 (HLA-A2^pos^, A24^pos^ and B7^neg^) and SUM190 (HLA-A2^neg^, A24^neg^, B7^neg^). 293T cells (HLA-A2^pos^, A24^neg^ and B7^neg^) were used as a FOXP3 negative cell line as shown in Figure 1A and representative immunoblot analysis (Figure 2A) of FOXP3 expression compared to positive control tonsil lysates and SUM149. As shown in the Figure 2B, FOXP3-specific effectors effectively lysed the FOXP3^pos^HLA-A2^pos^ targets, SUM149 but not the FOXP3^neg^HLA-A2^pos^ target 293, demonstrating antigen specificity. Notably, we did see low levels of lysis of the second IBC cell line, SUM190 (FOXP3^pos^HLA-A2^neg^), which presumably represents lysis mediated via HLA molecules that are shared between the donor used for this assay and the cell line. SUM190, derived from a primary tumor, is the only other human cellular model for IBC representing an ErbB2 overexpressing, ER^neg^ cell line. Of note is the fact that survivin-specific effector T cells did not lyse any of the targets, which corroborates our earlier observation that survivin is not a relevant target in these IBC cell lines [33]. Actin-specific effectors did not lyse any of the targets. As an additional level of control to demonstrate FOXP3-specificity of the effector T cells similar to our previous studies [40], [32], [43], we also examined the lysis of autologous DCs expressing FOXP3 by the effector T cells and demonstrate antigen-specific lysis of FOXP3-expressing DCs but not actin-expressing DCs (Figure 2C).

### Enhanced Expression of Anti-apoptotic Proteins and Resistance to FOXP3-specific T cell Mediated Tumor Cell Lysis

Since we were able to demonstrate lysis of FOXP3-expressing IBC cell lines using FOXP3-specific cytotoxic T lymphocytes (CTL), we next wanted to determine the effect of immunologic targeting of FOXP3 in rSUM149, an isogenic derivative of SUM149 with enhanced anti-apoptotic phenotype and resistance to apoptosis-inducing agents [31], [33], [44]. Using RNA-transfected DCs and T cells derived from PBMCs from an HLA-A2+ donor, we generated FOXP3-specific effectors *in vitro* and used them in a lytic assay with SUM149 and rSUM149 cells as targets. Indeed, as shown in the Figure 2D, we were able to reproduce the lysis of the SUM149 cells but did not observe lysis of the rSUM149 cells.

**Figure 3:**
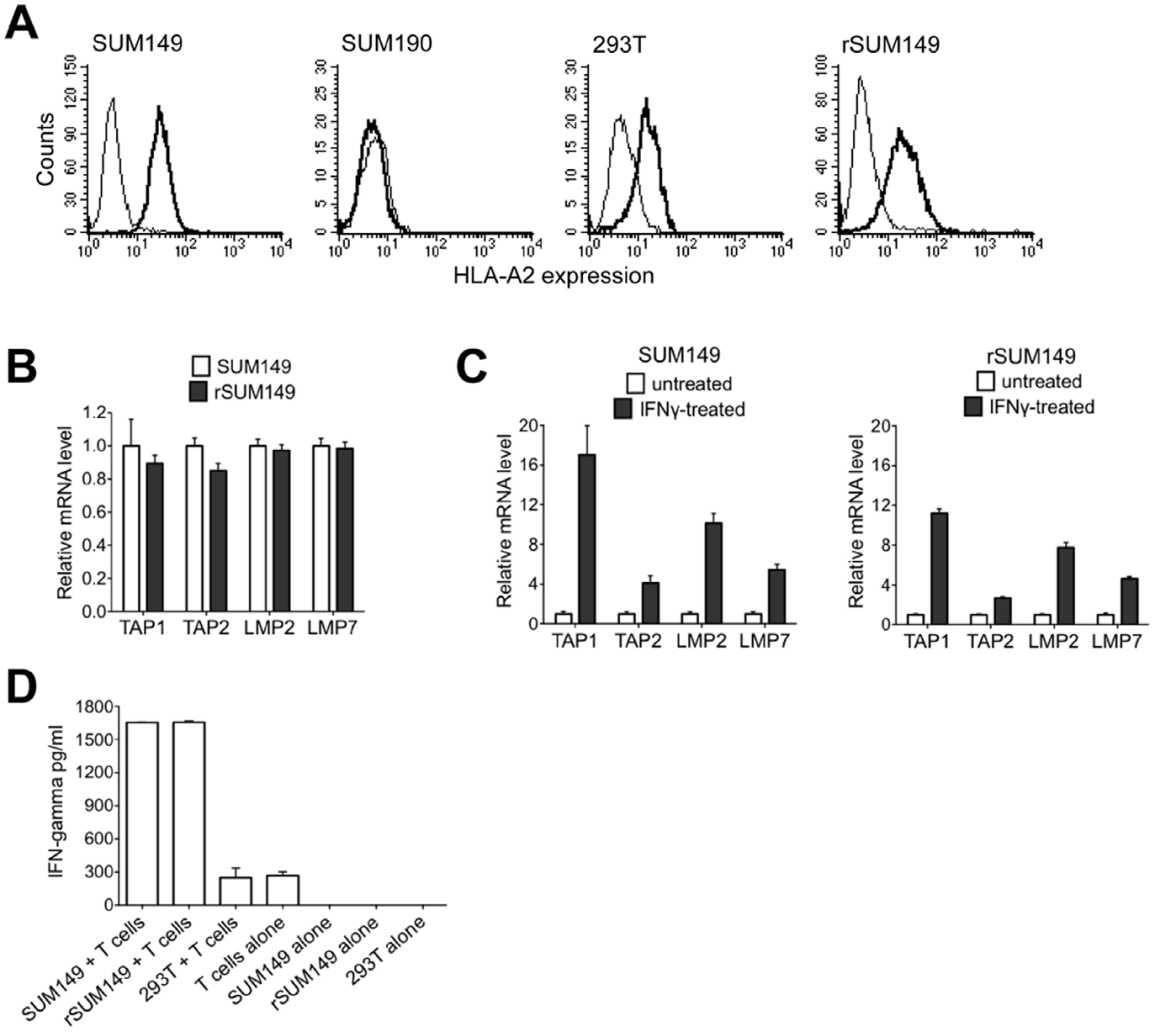
Evaluating MHC class I processing and presentation in SUM149 and rSUM149 cells. A. Cells used as targets in the CTL assay described in Figure 2 were analyzed for the cell surface expression of HLA-A2 class I molecule as indicated in the panel. The bold black histogram in each panel represents staining with the HLA-A2 specific antibody (clone BB7-2) versus the isotype control. **B.** Basal expression in the SUM149 and rSUM149 cells. The data shown is representative of replicated experiments analyzed using the ΔΔC(t) method and represented as relative expression (2^−ΔΔC(t)^). GAPDH was used as the normalization control. **C.** Expression after treatment with IFN-γ**.** The data shown is the average of replicated experiments analyzed using the ΔΔC(t) method and represented as relative expression (2^−ΔΔC(t)^). GAPDH was used as the normalization control with the untreated sample set to 1 and compared to the IFN-γ treated group. **D.** FOXP3-specific T cells were generated as described in Methods and Figure 2 above. T cells were allowed to rest overnight in RPMI medium with 10% FCS in the absence of cytokines. The following day, T cells were harvested and cocultured with target cells (SUM149, rSUM149 and 293T cells) at a 1∶1 ratio. T cells and target cells were maintained at 10^6^ cells/ml and a 100 µl of each cell suspension was added to a 96-well V-bottom tissue culture plate in triplicates and incubated at 37°C for 16 hours. Supernatant was harvested and IFN-γ secretion was measured using a human IFN-γ ELISA kit from eBioscience. Controls used are indicated in the figure.

To ascertain that the lack of cytolytic activity on rSUM149 cells was not due to dysregulation in MHC class I antigen processing mechanism, we evaluated MHC class I expression on these cells and determined that both cell lines express comparable levels of MHC molecule HLA-A2 by flow cytometry (Figure 3A). To further evaluate if MHC class I antigen processing components are different between SUM149 and rSUM149, we evaluated the expression of LMP2 and LMP7, the immunoproteasome subunits associated with class I antigen processing and TAP-1 and TAP-2, transporter-associated with class I antigen processing in the cell lines. As depicted in the Figure 3B we were not able to see any differences in these molecules in any of the groups tested. To further ascertain if there are any differences related to the MHC class I cell processing mechanism, we evaluated if the antigen presentation machinery is equally functional in the SUM149 and rSUM149 cells. The cells were therefore treated with interferon-gamma (IFN-γ), a known stimulator of the antigen processing machinery in cells. As indicated in the Figure 3C both cell lines equally and effectively responded to treatment with IFN-γ. To provide further evidence that SUM149 and rSUM149 cells present FOXP3-derived peptides in the context of MHC class I, we performed a recognition assay to demonstrate that both cell lines are capable of interacting with *in vitro* generated FOXP3-specific T cells. As shown in Figure 3D, both cell lines, SUM149 and rSUM149, are responsive to FOXP3-specific T cells and elicit IFN-γ secretion by the T cells as measured using a commercially available IFN-γ ELISA kit. Moreover, IFN-γ secretion was not observed when the T cells were co-incubated with the FOXP3-negative cell line, 293T. Collectively Figures 2 and 3 indicate that FOXP3 is a relevant immunological target in IBC, as indicated by the lysis of SUM149 cells and these results indicate the difference in lytic sensitivity in the SUM149 and the lapatinib-resistant rSUM149 IBC cells is not related to MHC class I expression in these cells or to the lack of MHC class I antigen processing machinery.

**Figure 4:**
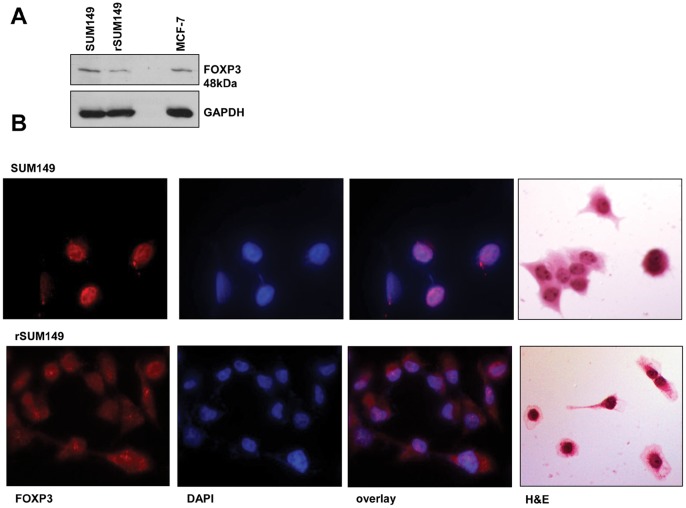
FOXP3 expression in breast cancer cells. A. Immunoblot analysis of FOXP3 and GAPDH is shown as loading control. **B.** Representative fluorescent microscopy images of cells stained with antibodies to FOXP3 and probed with Alexa Fluor® 568 goat anti-rat IgG. Each slide was counterstained with DAPI and the overlay for each is shown. Hematoxylin and eosin (H&E) staining is shown for morphology of each cell line.

**Figure 5:**
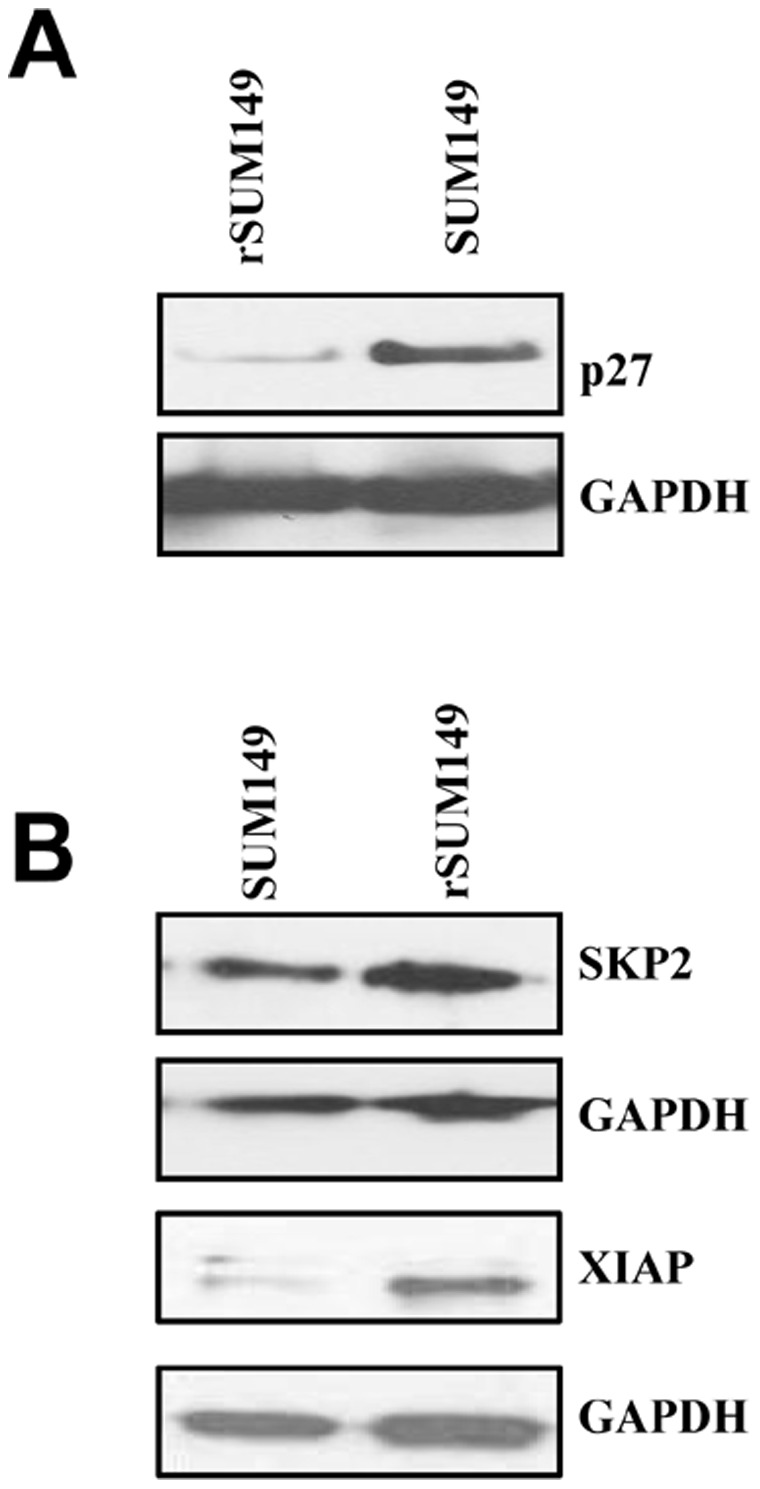
Expression of p27, XIAP and SKP2 in SUM149 and rSUM149 cells. Comparative expression of p27, an anti-apoptotic protein, XIAP, an inhibitor of apoptosis, and SKP2, an oncogene, in the GW58830-treated SUM149 and rSUM149 cells. GAPDH is shown as loading control in the immunoblots. **A.** Immunoblot analysis of expression of p27 **B.** Immunoblot analysis of expression of SKP2 and XIAP.

Furthermore, immunoblot analysis (Figure 4A) shows the FOXP3 protein expression (∼48 kDa) is similar in SUM149 and rSUM149 with MCF-7 cells as a positive control as described previously [41]. Interestingly, examination of the intracellular distribution of FOXP3 in SUM149 and rSUM149 cells revealed that in rSUM149 cells, cytoplasmic expression of FOXP3 was significantly higher compared to SUM149 cells (Figure 4B). Further, immunoblot analysis was carried out to evaluate the expression of p27, a pro-apoptotic cyclin D kinase inhibitor, which positively correlates with FOXP3 activity, and SKP2, a breast cancer oncogene known to be suppressed by FOXP3 transcriptional activity [21]. Data show induction of p27 (Figure 5A) and decrease in SKP2 levels (Figure 5B) in apoptosis-sensitive SUM149 cells. In contrast, in rSUM149 cells that exhibit an enhanced anti-apoptotic phenotype with significantly increased expression of XIAP (X-linked inhibitor of apoptosis protein), the most potent mammalian caspase inhibitor, SKP2 levels are sustained and there is no increase in p27 expression. In summary, the results indicate that FOXP3-specific T cells generated *in vitro* using human FOXP3 RNA-transfected DCs as stimulators efficiently lyse SUM149 cells, however increased anti-apoptotic signaling may contribute to tumor cell resistance to T-cell mediated lysis.

### FOXP3 Expression Correlates with a Low Recurrence Free Survival Rate in Breast Cancer Patients with a Triple Negative Phenotype

To further understand the clinical significance of FOXP3 expression in breast cancer cells, we characterized a recently developed gene expression dataset with 4010 breast tumor sets from NCBI Gene Expression Omnibus (GEO) database and a subset of the samples (n = 1372) that has recurrence-free survival data available [36].

**Figure 6:**
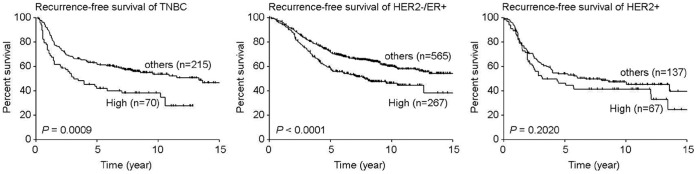
Recurrence-free survival in different breast subtypes according to *FOXP3* expression levels. Tick marks in Kaplan-Meier Estimates of recurrence-free survival indicate patients whose data were censored by the time of last follow-up or owing to death. *P* values were calculated using log-rank Mantel-Cox test.

From our combined gene expression data set, *FOXP3* expression was measured by two probe sets 221333_at and 221334_s_at. Using expression signal as continuous variable, we found each of these two probe sets was significantly associated with higher risk of recurrence (221333_at, *P = *2.13E−07; 221334_s_at, *P* = 8.07E−04; n = 1372, Cox-regression survival analysis). To further determine the correlation *FOXP3* gene expression and recurrence-free survival in different breast cancer subtypes, average expression signal of these two probe sets was used to represent *FOXP3* mRNA expression. We found higher expression of *FOXP3* (higher expression quartile, top 25%) was significantly associated with higher risk of recurrence in TNBC (P = 9.00E−04, n = 285, Kaplan-Meier Estimates survival analysis, Figure 6), and the median recurrence-free survival (RFS) in TNBC with high expression and others were 3.11 and 13.34 years, respectively. Similarly, poor RFS was also seen among patients with higher *FOXP3* expression in HER2−/ER+ breast cancer (*P*<0.0001, n = 832, Kaplan-Meier Estimates survival analysis, Figure 6), and the median RFS in tumors with high expression and others were 7.83 and 15.93 years, respectively.

## Discussion

We report herein for the first time that FOXP3 is expressed in cell line models of inflammatory breast cancer, an aggressive subtype of breast cancer with the worst survival outcomes. Although expression of the transcriptional protein FOXP3 specifically characterizes naturally occurring Tregs, it is now clear that FOXP3 is also expressed by many tumor cells including breast cancer cells, however its role is controversial and not clearly understood with its ability to act as a tumor suppressor in some cancer cells and a prognostic marker in others [45], [46]. Analysis of recurrence-free survival data from a subset of breast tumor gene expression data samples (n = 1372) from a collection of 23 datasets posted on the NCBI Gene Expression Omnibus (GEO) database, we demonstrated high expression of *FOXP3* significantly associated with higher risk of recurrence in TNBC. The role of FOXP3 in tumor cells is still unclear as it appears that expression of FOXP3 in tumor cells may be a mechanism of tumor-mediated immune suppression, similar to its function in Tregs but on the other hand, FOXP3 expression itself may suppress tumor growth, by repressing the expression of tumor oncogenes [18], [21]. Therefore, we determined if FOXP3 is an immunotherapeutic target in IBC using T cells stimulated with DCs transfected with FOXP3 RNA. This was based on our previous study demonstrating that immunization with DCs transfected with FOXP3 RNA, eliminates FOXP3-expressing Tregs, and enhances tumor immunity and vaccine-induced immune responses in a murine tumor immunotherapy model [32]. We demonstrate for the first time that FOXP3-specific T cells can effectively lyse a basal type, triple negative cell line SUM149 derived from IBC patient tumor.

Interestingly, our results show that an isogenic-derivative of SUM149 with enhanced anti-apoptotic phenotype is resistant to T cell lysis mediated by the DCs transfected with FOXP3 RNA. Successful elimination of tumors by cytotoxic T cells depends on TCR engagement of the antigen-class I MHC complex followed by conjugate formation, activation of downstream signaling events in T cells and delivery of the death signal by the cytotoxic T cell to the tumor cells. An immune evasion strategy that tumors use to avoid recognition is the down-regulation of antigen processing components that are critical for the establishment of the antigen-MHC complex on the cell surface [47], [48]. Examination of the components of the class I antigen processing pathway in SUM149 and rSUM149 cells revealed no difference in class I MHC expression, TAP expression or the expression of proteasome subunits. Moreover, SUM149 cells and rSUM149 cells were equally capable of eliciting IFN-γ production by FOXP3-specific T cells providing evidence that the cells do present FOXP3-derived peptides in the context of MHC class I molecules. These data indicate that the T cell recognition complex in rSUM149 cells is functional and is not the reason for lack of lysis of rSUM149 cells observed in in this study. We also observed that FOXP3 expression was more pronounced in the cytoplasmic compartment in rSUM149 cells. It is well established that FOXP3 nuclear expression is constitutive in human Tregs and nuclear localization is also observed in many normal and cancer cells wherein it acts as a transcription factor [12], [17], [18], [19]. Predominant cytoplasmic expression has been reported in pancreatic cancer cells [13] and in many breast specimens [49], [50] but the significance of cytoplasmic localization is not well understood. Whether cytoplasmic expression of FOXP3 is due to a mutation and/or causes a differential functional effect needs to be characterized.

A critical difference between the T cell lysis-sensitive SUM149 and insensitive rSUM149 is that the mechanism of acquired resistance in rSUM149 cells may be attributable to apoptotic dysregulation in these cells with concomitant over-expression of multiple anti-apoptotic proteins. rSUM149 has been identified to overexpress XIAP, the most potent mammalian caspase inhibitor [33] and these cells are also less sensitive to reactive oxygen inducing agents [31] and apoptosis inducing agents like TRAIL compared to parental SUM149 [44]. However, both cell lines express death receptors (DR4 and DR5) and we have observed that resistance to TRAIL is due to XIAP overexpression [44]. In addition, anti-apoptotic proteins, bcl2, survivin, p-NF-κB are not downregulated even with chronic exposure to lapatinib analog in the rSUM149 cells [33]. Interestingly, we have reported that rSUM149 have decreased FOXO3A compared to SUM149 cells [33] since it has been shown that increased expression of XIAP can negatively regulate c-jun NH2 terminal kinase-mediated FOXO3A upregulation thereby suppressing pro-apoptotic signaling mediated by FOXO3A. This is of relevance in the present study, since FOXO3A is another member of the FOXO family, which has the ability to drive FOXP3 expression [51], [52]. In the present study we also observed no induction of p27 or downregulation of XIAP and SKP2 in the rSUM149 cells compared to parental apoptotic-sensitive SUM149. The anti-apoptotic proteins that are expressed in the rSUM149 cells are therefore particularly relevant in immune evasion strategies used by the tumor cells to resist tumor-specific T cell-mediated killing. This is consistent with prior studies that have shown anti-apoptotic proteins lead to immune evasion and role for the IAPs in regulating T cell-dependent responses [53]–[55]. In fact, recent studies show that inhibitors of IAP proteins including XIAP can augment both prophylactic and therapeutic antitumor vaccines *in vivo* [56] and targeting XIAP degradation enhances granzyme and NK cell-induced cytolysis of target tumor cells [57]. Similarly, NF-κB inhibitor, bortezomib, has been shown to sensitize melanoma cells to adoptive CTL response [58]. Studies are underway to target the anti-apoptotic mechanism, in particular using XIAP antagonists in the rSUM149 cell and evaluate the role of these proteins in resistance to cytotoxic T cell mediated killing using siRNA-mediated knockdown of specific proteins.

Data presented in this study indicate that FOXP3 is a relevant immunotherapeutic target in IBC cells. On a cautionary note, we present data demonstrating that IBC cells that have acquired resistance due to presence of anti-apoptotic signaling may not be susceptible to FOXP3-targeting by cytolytic T cells. This study also indicates that the extrinsic apoptotic pathway deregulation may be one of the critical mechanisms, which renders tumors cells resistant to anti-tumor immune responses and needs to be studied further. These results suggest that effective elimination of chemotherapy-resistant IBC cells may require immunologic intervention coupled with strategies that target apoptotic dysregulation. In subsequent studies we will validate these findings by combining immunologic targeting of tumor antigens with specific inhibition of anti-apoptotic proteins using small molecule inhibitors.
